# Clinical Outcomes of Balloon-Occluded Versus Plug-Assisted Retrograde Transvenous Obliteration in the Treatment of Gastric Varices

**DOI:** 10.5334/jbsr.3881

**Published:** 2025-07-23

**Authors:** Youngjong Cho, Sung-Joon Park, Hyoung Nam Lee, Sangjoon Lee, Seung Soo Kim

**Affiliations:** 1 Department of Radiology, University of Ulsan College of Medicine, Gangneung Asan Hospital, Gangneung, Republic of Korea; 2 Department of Radiology, Korea University College of Medicine, Korea University Ansan Hospital, Ansan, Republic of Korea; 3 Department of Radiology, Soonchunhyang University College of Medicine, Cheonan Hospital, Cheonan, Republic of Korea; 4 Seoul Samsung Clinic, Cheonan, Republic of Korea

**Keywords:** esophageal and gastric varices, portal hypertension, balloon occlusion, sodium tetradecyl sulfate, sclerosing solutions

## Abstract

*Purpose:* To compare balloon-occluded retrograde transvenous obliteration (BRTO) using sodium tetradecyl sulfate and plug-assisted retrograde transvenous obliteration (PARTO), for treating symptomatic gastric varices.

*Materials and methods:* A retrospective review of 51 consecutive patients (age: 63.7 ± 12.1 years; male: 72.6%) who underwent retrograde transvenous obliteration for gastric varices between June 2018 and July 2023 was conducted. Patients underwent BRTO (*n* = 26) or PARTO (*n* = 25) according to the preference of the attending interventional radiologist. The primary endpoint was complete obliteration. Secondary endpoints included technical and clinical success, post-embolization syndrome (PES), complications, and recurrent bleeding.

*Results:* Technical success was achieved in 100% of BRTO group and 96% of PARTO group. Clinical success was achieved in all patients with technical success. One major complication in the PARTO group was due to shunt rupture during sheath passage, requiring fluid resuscitation and blood transfusion. In the BRTO group, there was a minor complication involving a balloon rupture, but the patient remained asymptomatic. PES was more frequent in the PARTO group (56%) compared to the BRTO group (23.1%) (*p* = 0.034). The median follow-up duration was 7 months. Complete obliteration rate was significantly higher in the BRTO group (100%) than in the PARTO group (80%) (*p* = 0.023). Recurrent bleeding occurred in one patient with remnant varix in the PARTO group.

*Conclusions:* Both techniques achieved high technical and clinical success rates in the treatment of symptomatic gastric varices. However, BRTO demonstrated higher complete variceal obliteration and lower PES, suggesting a clinical advantage over PARTO.

## Introduction

Gastric varices represent a serious complication of portal hypertension, often requiring immediate and effective intervention to prevent life-threatening bleeding. Balloon-occluded retrograde transvenous obliteration (BRTO) is an interventional radiologic procedure used for treating gastric varices with a gastrorenal shunt, which has a well-established safety and efficacy record [1]. However, a notable drawback of BRTO is the need for prolonged balloon retention, which may extend beyond 24 h, leading to increased procedure time and patient discomfort. There remains a risk of complications with sclerosing agents, but it has been substantially reduced with the introduction of sodium tetradecyl sulfate (STS) [2–4].

In contrast, plug-assisted retrograde transvenous obliteration (PARTO) has emerged as a novel alternative, eliminating the need for balloon retention and sclerosing agents [5]. PARTO tends to have shorter procedure times than BRTO, potentially reducing overall procedural complexity and improving patient experience, making it an attractive option for many clinicians. Nevertheless, PARTO has not been able to completely replace BRTO, partly due to technical difficulties, such as the need for insertion of a large guiding sheath into the gastrorenal shunt for plug placement [6]. Furthermore, there have been instances of vascular plug migration, raising concerns about long-term stability [7].

PARTO has been increasingly adopted and accepted in clinical practice, but only a few studies have compared its performance to BRTO [4]. The trade-offs between convenience, efficacy, and safety across these two techniques remain incompletely understood, and existing evidence does not adequately guide clinicians in selecting the optimal treatment for each patient. The purpose of this study is to compare the efficacy and safety of BRTO and PARTO in treating symptomatic gastric varices, thereby contributing to a more comprehensive understanding of the advantages and limitations of each technique.

## Materials & Methods

The Institutional Review Board of a tertiary care hospital approved this retrospective study and waived written informed consent for using clinical and imaging data. Written informed consent for interventional procedures was obtained from all patients.

### Study design

From June 2018 to July 2023, 51 consecutive patients (mean age: 63.7 ± 12.1 years; 72.6% male) with gastric varices with acute upper gastrointestinal bleeding or a history of bleeding were treated with retrograde transvenous obliteration (RTO) via gastrorenal shunts. These patients underwent BRTO (*n* = 26) or PARTO (*n* = 25). The choice of RTO technique was left to the preference of the attending interventional radiologist, considering the shunt diameter from the pre-procedure CT and available instruments at the hospital. Medical records, including data on radiologic studies, were used to collect demographic information, presence of hepatocellular carcinoma (HCC), Child–Pugh classification, the model for end-stage liver disease (MELD) score, access route, instruments employed in the procedure, grade of gastric varices, the necessity for collateral embolization, fluoroscopy time, complication, post-embolization syndrome (PES), obliteration of gastric varices, and recurrent bleeding.

### Definition

The primary endpoint was complete obliteration. Secondary endpoints included technical and clinical success, PES, complications, and recurrent bleeding. Technical success was defined as positioning an occlusion balloon or vascular plug within the gastrorenal shunt, followed by embolic or sclerosant material infusion. Clinical success was defined as cessation of active bleeding, clinically and on endoscopy and complete or partial thrombosis of gastric varices on CT. Major and minor complications within one month of the procedure were classified according to the guidelines of the Society of Interventional Radiology Standards of Practice Committee [8]. PES was not considered a complication. It was defined as a separate condition: fever with or without abdominal pain that developed within three days of the procedure without evidence of infection [9–11]. Complete obliteration was defined as the total eradication of targeted gastric varices on a follow-up CT. Recurrent bleeding was defined as hematemesis or melena, along with endoscopic confirmation of bleeding originating from gastric varices.

### Procedures

All patients underwent pre-procedural evaluations, including medical history, physical examination, and laboratory testing to assess liver function and coagulation status. Upper gastrointestinal endoscopy was performed to confirm the presence and type of varices. Contrast-enhanced CT was used to assess the patency of the portal vein, identify gastrorenal shunt, and evaluate the size and anatomy of the gastric varices and related collateral veins. The presence of ascites, splenomegaly, or signs of advanced liver disease was also evaluated on imaging.

All procedures were performed by one of the two institutional, board-certified fellowship-trained interventional radiologists. Conscious sedation was achieved with intravenous pethidine hydrochloride (25 mg/0.5 mL, Je-il Pharmaceutical, Taegu, Korea) mixed with 100 mL of normal saline. The puncture site was sterilized, and 2% lidocaine (20 mg/mL, Huons, Seoul, Korea) was used for local anesthesia. The right common femoral vein was punctured initially with a micropuncture kit (Merit S-MAK™, Merit Medical, South Jordan, Utah, USA) under ultrasound guidance. When femoral access failed due to unfavorable angulation for negotiating the gastrorenal shunt, the right internal jugular vein was accessed instead.

### BRTO

After the guiding sheath (6–7-Fr Flexor Check-Flo; Cook Medical, Bloomington, IN, USA) was inserted into the gastrorenal shunt or left renal vein, an occlusion balloon catheter (5.5-Fr Fogarty; Edwards Lifesciences, Irvine, USA, 6-Fr TufTex; LeMaitre, Burlington, Massachusetts, USA) was advanced over the guidewire (Radifocus; Terumo, Tokyo, Japan). The balloon was positioned at the narrowest part of the shunt and inflated to block the outflow of gastric varices. The anatomic type of gastric varices was determined based on retrograde venography findings [12]. When indicated, collateral embolization was performed using a 1.9-Fr coaxial microcatheter (Carnelian SI; Tokai Medical Products Aichi, Japan) through the occlusion balloon catheter. A sclerosing agent was injected through the occlusion balloon catheter. The angiographic endpoint was the minimal filling of the afferent vein. The sclerosing agent consisted of a 2:1:3 mix of 3% STS (Fibrovein; STD Pharmaceutical, Hereford, UK), iodized oil (Lipiodol; Guerbet, Paris, France), and room air. The occlusion balloon was removed after 3–5 h of sclerosing time.

### PARTO

After the guiding sheath (6–9-Fr Flexor Check-Flo; Cook Medical, Bloomington, IN, USA) was advanced into the gastrorenal shunt, a vascular plug (8–18 mm Amplatzer Vascular Plug II, St. Jude Medical, St. Paul, MN, USA) was introduced alongside the safety guidewire (Radifocus; Terumo, Tokyo, Japan) and then deployed at the widest portion of the shunt. The size of the vascular plug was selected to be approximately 20% larger than the narrowest portion of the shunt. Over the safety guidewire, a 4-Fr catheter (Berenstein; Cook Medical, Bloomington, IN, USA) was advanced through the gap between the vascular plug and the shunt wall. The plug was then pulled downward to position the narrowest part of the shunt. Embolization was performed using a hand-cut gelatin sponge (Spongostan; Ferrosan Medical Devices, Søborg, Denmark) with contrast media through the 4-Fr catheter. Additional embolization was attempted at collateral veins that were not spontaneously occluded, using a 1.9-Fr coaxial microcatheter (Carnelian SI; Tokai Medical Products Aichi, Japan) through the 4-Fr catheter. The angiographic endpoint was the same as that used in BRTO. The 4-Fr catheter was removed, and the vascular plug was detached.

### Follow-up

A follow-up endoscopy was performed 4–5 weeks after the procedure to assess for residual varices and possible re-treatment. A follow-up CT scan was conducted 2–3 months later to confirm the complete obliteration of gastric varices. Further follow-up evaluations were determined based on individual patient needs and the referring physician’s judgment.

### Statistics

The comparative analysis employed a two-sample *t*-test or Wilcoxon rank-sum test for continuous variables and a chi-squared test or Fisher’s exact test for categorical variables. A *p*-value < 0.05 was considered statistically significant. All statistical analyses were executed using R version 3.6.3 software (Foundation for Statistical Computing, Vienna, Austria).

## Results

No significant differences were observed between the two groups in baseline characteristics, including age (*p* = 0.291), gender (*p* = 0.392), etiology of liver disease (*p* = 0.569), presence of HCC (*p* = 0.291), Child–Pugh classification (*p* > 0.99), and MELD score (*p* = 0.147), as summarized in Table 1. Details of the procedure are summarized in Table 2. Conversion to jugular vein access was more common in the PARTO group (16%) than in the BRTO group (3.9%), but not statistically significant (*p* = 0.191). There was a significant difference in the diameter of the used sheath between the two groups (*p* < 0.001), with 8-Fr and 9-Fr sheaths used exclusively in the PARTO group. Collateral embolization was more frequently required in the BRTO group (53.9%) than in the PARTO group (16%) (*p* = 0.011). There was no significant difference in fluoroscopy time between the groups (*p* = 0.509).

**Table 1:** Patient characteristics.

PARAMETER	ALL	BRTO	PARTO	*P*-VALUE
	(*N* = 51)	(*N* = 26)	(*N* = 25)	
Age (yr), mean ± SD	63.7 ± 12.1	61.9 ± 12.4	65.5 ± 11.6	0.291
Gender (male), *n* (%)	37 (72.6%)	17 (65.4%)	20 (80%)	0.392
Underlying liver disease, *n* (%)				0.569
HBV cirrhosis	16 (31.4%)	10 (38.5%)	6 (24%)	
HCV cirrhosis	1 (2%)	0 (0%)	1 (4%)	
Alcoholic cirrhosis	26 (51%)	12 (46.2%)	14 (56%)	
Other	8 (15.7%)	4 (15.4%)	4 (16%)	
Concomitant HCC, *n* (%)	10 (19.6%)	7 (26.9%)	3 (12%)	0.291
Child–Pugh classification, *n* (%)				> 0.99
A	29 (56.9%)	15 (57.7%)	14 (56%)	
B	19 (37.3%)	9 (34.6%)	10 (40%)	
C	3 (5.9%)	2 (7.7%)	1 (4%)	
MELD score, median (IQR)	8 (7.5, 11)	8 (6.3, 9.8)	9 (8, 11)	0.147
Follow-up (mo), median (IQR)	7 (3.5, 16.9)	6 (3.8, 9.9)	13.7 (3.5, 17.6)	0.327

BRTO = balloon-occluded retrograde transvenous obliteration; PARTO = plug-assisted retrograde transvenous obliteration; HBV = hepatitis B virus; HCV = hepatitis C virus; HCC = hepatocellular carcinoma; MELD = Model for End-stage Liver Disease, IQR = interquartile range.

**Table 2:** Details of the procedure.

PARAMETER	ALL	BRTO	PARTO	*P*-VALUE
	(*N* = 51)	(*N* = 26)	(*N* = 25)	
Access route, *n* (%)				0.191
Right common femoral vein	46 (90.2%)	25 (96.2%)	21 (84%)	
Right internal jugular vein	5 (10%)	1 (3.9%)	4 (16%)	
Sheath diameter, *n* (%)				< 0.001*
6-Fr	28 (54.9%)	13 (50%)	15 (60%)	
7-Fr	14 (27.5%)	13 (50%)	1 (4%)	
8-Fr	7 (13.7%)	0 (0%)	7 (28%)	
9-Fr	2 (3.9%)	0 (0%)	2 (8%)	
Collateral embolization, *n* (%)	18 (35.3%)	14 (53.9%)	4 (16%)	0.011*
Fluoroscopy time (m), median (IQR)	36 (25, 47.5)	34 (21.3, 53.5)	36 (27, 40)	0.509

*p < 0.05 means statistical significance.

BRTO = balloon-occluded retrograde transvenous obliteration; PARTO = plug-assisted retrograde transvenous obliteration, IQR = interquartile range.

In the BRTO group, 5.5 mm (*n* = 13) and 6 mm (*n* = 13) occlusion balloon catheters were employed for the procedure. The BRTO group had the following grades of gastric varices based on retrograde venography: grade 1 (*n* = 7), grade 2 (*n* = 6), grade 3 (*n* = 7), and grade 4 (*n* = 6). In the PARTO group, vascular plugs of the following sizes were utilized: 8 mm (*n* = 6), 10 mm (*n* = 4), 12 mm (*n* = 6), 14 mm (*n* = 4), 16 mm (*n* = 2), and 18 mm (*n* = 2). In both groups, various embolic materials were used for collateral embolization: gelatin sponge (*n* = 4), glue-lipiodol mixture (*n* = 5), micro-coil (*n* = 5), and multiple agents (*n* = 4).

Clinical outcomes are summarized in Table 3. Technical success was achieved in all cases in the BRTO group and in 96% of cases in the PARTO group (*p* = 0.49). Clinical success was achieved in all cases where technical success was obtained. There were no significant differences in major (*p* = 0.49) or minor (*p* > 0.99) complication rates between the two groups. One technical failure occurred in the PARTO group due to shunt rupture during sheath insertion, resulting in a major complication. The patient developed hemodynamic instability due to bleeding from the rupture site, requiring intravenous fluid resuscitation and blood transfusion, and was subsequently treated with endoscopic sclerotherapy after stabilization. In the BRTO group, there was one minor complication involving balloon rupture after 3 h of balloon inflation time. The patient exhibited no clinical signs or symptoms of pulmonary embolism and was discharged three days after the procedure without further management.

**Table 3:** Clinical outcomes.

PARAMETER	ALL	BRTO	PARTO	*P*-VALUE
	(*N* = 51)	(*N* = 26)	(*N* = 25)	
Technical success, *n* (%)	50 (98%)	26 (100%)	24 (96%)	0.49
Clinical success, *n* (%)	50 (98%)	26 (100%)	24 (96%)	0.49
Major complication, *n* (%)	1 (2%)	0 (0%)	1 (4%)	0.49
Minor complication, *n* (%)	1 (2%)	1 (3.9%)	0 (0%)	> 0.99
Post-embolization syndrome, *n* (%)	20 (39.2%)	6 (23.1%)	14 (56%)	0.034*
Complete obliteration, *n* (%)	46 (90.2%)	26 (100%)	20 (80%)	0.023*
Recurrent bleeding, *n* (%)	1 (2%)	0 (0%)	1 (4%)	0.49

*p < 0.05 means statistical significance.

BRTO = balloon-occluded retrograde transvenous obliteration; PARTO = plug-assisted retrograde transvenous obliteration.

**Figure 1:**
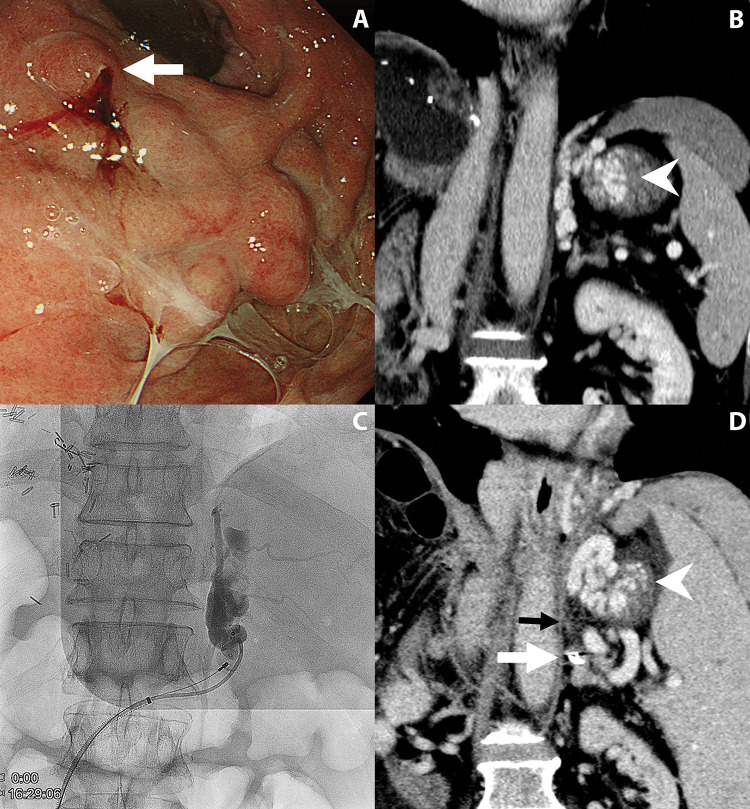
A 62-year-old man who underwent plug-assisted retrograde transvenous obliteration. **A.** Endoscopic examination shows gastric variceal bleeding (arrow). **B.** Coronal contrast-enhanced CT scan reveals a large and tortuous varix (arrowhead) at the gastric fundus. **C.** Fluoroscopic image shows a vascular plug deployed at the gastrorenal shunt. After injection of gelatin sponge slurry, gastric varices are only partially opacified. Selective catheterization of collaterals failed due to severe tortuosity. **D.** Follow-up CT scan shows an obliterated gastrorenal shunt (black arrow) with a vascular plug (white arrow) and residual gastric varices (arrowhead).

PES was significantly more common in the PARTO group (56%) compared to the BRTO group (23.1%) (*p* = 0.034). Complete obliteration of gastric varices was achieved in 90.2% of cases, with a significantly higher rate in the BRTO group (100%) compared to the PARTO group (80%) (*p* = 0.023). Of the five patients with incomplete obliteration, one underwent endoscopic band ligation (Figure 1), another underwent endoscopic sclerotherapy, and the remaining three patients were followed without additional interventions. Recurrent bleeding occurred in one patient with remnant varix in the PARTO group, while none occurred in the BRTO group (*p* = 0.49). The median follow-up period was 7 months (range, 3.5–16.9 months).

## Discussion

BRTO is an effective alternative to the transjugular intrahepatic portosystemic shunt for treating bleeding gastric varices with favorable anatomy (e.g. accessible and occludable gastrorenal shunt) [1, 13]. Recently, various modified BRTO techniques, including PARTO, have gained attention due to their potential for improved efficacy and safety [14]. This study aimed to compare the clinical outcomes of two RTO techniques, BRTO and PARTO, in treating symptomatic gastric varices. A total of 51 patients underwent either BRTO or PARTO over a five-year period, and clinical outcomes were assessed in terms of technical and clinical success, complete obliteration, recurrent bleeding, and safety profiles.

Both techniques demonstrated high technical and clinical success rates with low complication rates, indicating their viability as treatments for gastric varices. When comparing procedural characteristics, PARTO required a larger guiding sheath than BRTO because it needed both a plug delivery system and an extra catheter. This made PARTO technically more challenging, particularly when positioning the larger sheath in the shunt for plug deployment. A major complication of PARTO was also associated with the guiding sheath passage. In addition, PARTO required more conversions to right internal jugular access than BRTO, although the difference did not reach statistical significance. These findings align with previous studies indicating that anatomical factors are a significant predictor of technical failure of PARTO [6].

Notably, PARTO offers the advantage of requiring less collateral embolization than BRTO, as small collateral veins can be controlled without selective catheterization. Despite these procedural differences, there was no significant difference in fluoroscopy time between the two groups. In contrast, BRTO requires an extended balloon inflation time for sclerotherapy after the procedure. Based on previous studies, balloon inflation was maintained for at least 3 h in this study to prevent technical failure [15]. Balloon rupture is a rare but significant complication specific to BRTO, which can lead to technical failure and pulmonary embolism. In this study, the use of alternative occlusion balloon catheters instead of dedicated BRTO catheters may have contributed to balloon rupture [16]. Although the patient did not report any clinical symptoms related to this issue, post-procedural monitoring remains crucial to ensure proper management [17].

The PARTO group showed a higher occurrence of PES. The pathophysiology of PES after RTO might differ from other embolization procedures because RTO does not induce direct organ infarction or necrosis [9]. One possible cause of PES after RTO could be a large clot burden intentionally generated during the procedure [18]. Research studies comparing PES across various RTO techniques are limited [4]. The higher incidence of PES in the PARTO group might be due to reactions to metallic foreign bodies or the use of substantial gelatin sponge slurry. Gelatin sponges have been associated with more frequent PES than microspheres in previous studies [19, 20]. In addition, STS foam has been found to reduce the dose of sclerosant in BRTO compared to ethanolamine oleate, which may explain the lower incidence of PES [2].

Complete obliteration of gastric varices is crucial for preventing rebleeding. This study found that the BRTO group had a higher rate of complete obliteration than the PARTO group, consistent with findings from a previous study by Kim et al. [4]. This discrepancy is likely explained by differences in embolization mechanisms and collateral management. Sclerotherapy chemically stimulates endothelial damage, denudation, thrombosis, and collagen fiber contraction, ultimately transforming the vessels into fibrous strands [21]. The temporary obstruction and simple thrombosis caused by the gelatin sponge can be recanalized if collateral blood flow is present [22]. The irregular shape of large gelatin sponge particles may lead to insufficient or proximal embolization of collateral veins. In contrast, BRTO provides a comprehensive view of the complex variceal anatomy, guiding the entire procedure [23]. Although time-consuming, selective collateral embolization appears to be effective in preventing recanalization.

The current study has several limitations, including its retrospective and observational design, which might have introduced selection and information bias. The choice of each technique was subject to the discretion of the attending interventional radiologist. Equipment availability in the hospital at the time of the procedure might have influenced these decisions. In addition, baseline characteristic matching was not performed. However, the two groups had no significant differences in comparative analysis. As the follow-up period for each patient was variable, evaluating the long-term outcome was challenging.

In conclusion, both techniques achieved high technical and clinical success rates in the treatment of symptomatic gastric varices. However, BRTO demonstrated a higher rate of complete variceal obliteration and a lower incidence of PES, suggesting a clinical advantage over PARTO despite its longer procedure time.
